# Characteristic Variation of Electromechanical Delay After the Botulinum Toxin Injection in Spastic Biceps Brachii Muscles

**DOI:** 10.3389/fneur.2021.789442

**Published:** 2022-02-10

**Authors:** Sourav Chandra, Babak Afsharipour, William Z. Rymer, Nina L. Suresh

**Affiliations:** ^1^Department of Physical Medicine and Rehabilitation, Northwestern University, Chicago, IL, United States; ^2^Arms and Hand Laboratory, Shirley Ryan Ability Lab, Chicago, IL, United States; ^3^Department of Biomedical Engineering, University of Alberta, Edmonton, AB, Canada

**Keywords:** botulinum toxin, electromechanical delay, spasticity, surface electromyography, voluntary contraction

## Abstract

The objective of this study was to characterize the effects of intramuscular botulinum toxin (BT) injections on the electromechanical delay (EMD) in spastic human biceps muscles. The EMD is calculated as the time lag between the muscle activation onset, as recorded from the surface electromyogram (sEMG), and the onset of recorded force. In a cohort of chronic stroke survivors, we compared the computed EMD derived from the spastic (injected) biceps brachii with that from the contralateral muscle. Eight participants were tested before and up to 3 months after a BT injection. At each session, participants followed an isometric trapezoidal force trajectory at 50 and 30%, respectively, of the tested maximum voluntary contraction (MVC). Joint force and sEMG signals were recorded as well. The number of zero crossings (ZC) of the sEMG during the steady-state portion of the task was also computed. The EMD post-BT was found to increase by 64 ± 10% (at 50% MVC) and 93 ± 18% (at 30% MVC) when compared to pre-BT values, while the number of sEMG-ZC, the mean MVC values, and the force-EMD slope exhibited striking reductions. These parameters, calculated on the contralateral side, remained relatively constant across sessions, with the EMD significantly lower and the MVC values much higher. We discuss potential contributing factors to an increase in EMD values on the affected side, both pre- and post-BT. The observed co-variation across sessions of the increased EMD values with the decreased ZC estimates, a surrogate of motor outflow, and, potentially, more compliant muscle fascicles suggests that the altered motor unit (MU) behavior contributes, at least in part, to the delayed force production.

## Introduction

The process of tension generation in the neurally activated muscle fiber begins at the neuromuscular junction, followed by a cascade of electromechanical events (1). There is a finite lag after the neural activation of muscle fibers before there is detectable force generation by the muscle-tendon system (2, 3). In humans, this lag is routinely computed as the time difference between the onset of the sEMG and the detected force, and it has been designated as the electromechanical delay (4). The value of the EMD can be used as a useful biomarker for assessments of neuromuscular atrophy (5) and as a potential internal disruption of the muscle architecture (6).

In general, the electromechanical delay, which is partly a function of a muscle's rate of force generation, depends on two major factors: (a) the number of recruited motor units, and (b) the electro-mechanical properties of the muscle-tendon unit (MTU). The latter is dominated by intrinsic stiffness of the muscle and tendon (2). Neural commands determine the number of recruited MU during a muscle contraction (7). Once an MU is recruited, this event is followed by several finite duration processes in the fiber contributing to the electromechanical delay (i.e., synaptic transmission, excitation-contraction coupling, and MUAP propagation) (8–10). Additional contributions to the delay came from the presence of (passive) elastic elements that are in series with the contractile elements. Intramuscular Botulinum toxin injections are increasingly used to treat focal spasticity, and are known to affect both neural and non-neural properties that are subsequent to injection in spastic muscles (11).

Botulinum toxin reduces hypertonia and hyperreflexia by means of the chemodenervation at the neuromuscular junction (NMJ) (12). Generally, the period between successive BT injections chosen by a managing physician is ~10–12 weeks with the survivors of chronic stroke, and the maximum effect is observed within 1 month after the injection (13). While BT reduces spasticity due to cholinergic blockade of the NMJ, it also alters the neuromuscular control during the contraction (14) and also affects the passive mechanical properties of the muscle, as well as the MTU (15). This includes reduction of the passive stiffness and the muscle fiber diameter after the BT injection (16).

Haubruck et al. have studied the passive biomechanical properties of the muscle-tendon unit in the healthy mouse gastrocnemius muscle during stretching, and have reported a decrease of stiffness by 25% after the botulinum toxin (17). Howren et al. have reported a significant reduction of the muscle-tendon ratio after 6 weeks of botulinum toxin injection by monitoring the changes in length of the gastroc/soleus, as well as the Achilles tendon unit based on ultrasound images in 36 human clubfoot patients (18). The changes in the mechanical properties of the muscle and the MTU (i.e., reduction in muscle stiffness and increase slackness of MTU) could impair the overall force production capacity during the muscle stretch, thereby influencing the delay between the muscle activation and the resulting force generation. The botulinum toxin (BT) is known to impact various factors during the muscle contraction process that could affect the EMD, such as altering the neural control due to chemodenervation (19) and the reduced synaptic transmission (20), thereby affecting the excitation-contraction coupling (15). However, the alterations in the EMD values after a BT injection have not been studied in spastic human participants. It is also unclear, how long the changes in passive mechanical properties and neural control last, and whether these effects continue to affect the EMD.

In the last few decades, the EMD values have been quantified by several researchers in humans under several conditions. The EMD value during a voluntary contraction of the biceps brachii (abbreviated to biceps from hereon) in intact subjects was reported to be 75 ms (21) and 80 ± 20 ms (22). Historically, there has been a wider range in the EMD values, as reported by several researchers. The EMD has been conventionally reported to vary from 30 ms to more than 100 ms in biceps of healthy individuals during a voluntary isometric contraction (23). A similar range of EMD values (30–130 ms) was also reported by other groups (4). A fairly large variation of the EMD is attributed to several contributing factors as summarized earlier. The discrepancy of the EMD values in the literature may also be related to the differences in the adapted methodological approach to the onset detection. The onset detection method for sEMG, and force/torque signals, indeed, form the quintessential attribute (24).

Lacourpaille et al. have studied the human biceps muscle during electrically evoked contraction and have found that 56% of the EMD is contributed by the muscle during an active force generation, while the rest is by the MTU (25). Mörl et al. have estimated a minimum EMD for an optimal length of MTU in a simulation study (26). While the mechanical properties are important, the influence of a neural control on the EMD was also found to be significant as indicated in a recent study by our group (27).

The number of turns in an sEMG signal has been used by several researchers as a rough measure of the MU activity during a voluntary contraction of the muscle as shown experimentally (28). During the contraction, the number of ZCs in the sEMG was found to be proportional to the number of recruited MUs, especially at lower force levels (29). Zhou et al. used a simulation-based study to show that ZC can be used to accurately resemble the number of recruited MU (30); experimental studies on human subjects have also reported the influence of the recruited MU on the ZC (31).

Although the clinical consequences of BT have been widely investigated (32, 33), the effect of BT on the EMD has never been experimentally explored in humans. Our group has reported earlier the influence of the mechanical properties of the muscle on EMD. In this context, we hypothesize that the altered force generation, due to a partial denervation of MU after BT injection, influences the EMD during the force generation. This will potentially provide means of non-invasive measurements of the muscle contraction efficiency during the time period after a BT injection.

Accordingly, the objective of the current study is to characterize and to understand the effects of BT on the electromechanical delay by computing the EMD values during the isometric voluntary contractions over a period of several weeks, in the course of multiple sessions in the post-injection period. We have measured the force and the sEMG during the isometric voluntary contraction in eight stroke-affected individuals who were injected with BT, as a part of their standard clinical care in their biceps muscle. As a surrogate measure of an MU recruitment, we also computed the number of ZCs or “turns” in the respective sEMG signal over time.

## Methods

### Subject Details

We report data from eight survivors of chronic stroke, who were studied before (Pre) and after (Post-W2) a BT injection, to assess the changes in the EMD subsequent to an intramuscular BT injection on the affected spastic side only. Six of the eight tested subjects were able to continue testing to the mid-segment (Post-W6), and 5 subjects were tested until the third month (until Post-W12). We have tested both the BT injected spastic biceps and the contralateral arm at different submaximal contraction levels. Details of our tested cohort are summarized in Table 1.

**Table 1:** Subject details, time of stroke, injection dosage, clinical scores before the injection and the number of months they were tested after the injection.

**Subject**	**Sex**	**Age**	**Impaired side**	**Post stroke (years)**	**BT dosage (human unit)**		**Modified Ashworth Scores at pre-session**	**Fugl-Meyer Scores at pre-session**	**Months tested after injection**
					**Biceps Brachii**	**Brachio-radialis**			
B1	F	56	Right	7	**50**	0	1+	17	3
B2	F	40	Center	10	**80**	30	1+	19	3
B3	M	60	Right	5	**50**	0	2	11	3
B4	F	43	Center	4	**40**	0	3	23	3
B5	M	55	Center	5	**100**	0	1+	20	3
B6	F	30	Center	12	**200**	0	3	14	2
B7	M	62	Right	7	**50**	0	1+	19	1
B8	M	84	Center	6	**50**	0	3	14	1

*Modified Ashworth Scores indicates the levels of spasticity and Fugle-Meyer Scores indicates the level of motor recovery for each subject. The bold values represent the dosage in the muscle recorded (biceps brachii)*.

We collected the experimental and clinical data at each session from stroke survivors, across a maximum of 3 months per subject. Approximately, there were over 45 total experimental recording sessions in both stroke-affected and contralateral arm muscles. The contralateral recordings were performed in separate sessions using the same protocol. The recordings from the contralateral side were used for comparative purposes as normative (non-injected) experimental data. Details of our experimental process, data collection, and analysis are summarized in the following section. All participants gave an informed consent *via* protocols approved by the Institutional Review Board under the Office for the Protection of Human Subjects at the Northwestern University (IRB No-STU00200242).

### Apparatus/Instrumentation

Two types of sEMG electrodes were used during the experiment. A single differential surface electrode (SD-sEMG) (Bagnoli, Delsys, Inc. Natick, USA) was placed over the muscle belly in the distal region of biceps, along the fiber direction. The single differential sEMG preamplifier has a bandwidth of 20–450 Hz for surface signals, amplified by a factor of 1,000, and sampled with a band-pass filter of 20–450 Hz (CMRR > 92 dB, input noise < 1.2 μV and impedance of 1,015 Ω in parallel with 0.2 pF). The force and single differential EMG signals were digitized at a rate of 2 kHz (Power 1401, CED Inc., Cambridge, UK). Force and sEMG signals were then collected and were visualized online using a software program from CED, Spike2 (version 7), and were stored on a personal computer for later analysis. The synchronized data allowed an accurate determination of the onset of sEMG in relation to the onset of torque production (34).

In order to analyze the zero crossings of the sEMG, we utilized recordings from a novel 4 channel sensor array (SA-sEMG) electrode (35). The sensor array has 5 sharp electrodes (evenly distributed within an area of 5 × 5 mm) and simultaneously records four channels of sEMG. The signals are then sampled at a much higher rate (20 kHz), acquired through a dedicated amplifier (dEMG, Delsys, Inc., Natick, USA), visualized, and stored using a computer-controlled software program. The sensor array electrode has an identical size and a footprint to the single differential electrode, and it was placed in the proximal side of the biceps muscle aligned with the SD-sEMG electrodes. We measured and recorded the boundaries of the electrode location from bony references such as acromion, cubital fossa, medial, and lateral epicondyles of the humerus at the first session of data collection (pre-injection), and used these same values for the electrode placement to preserve the consistency of electrode placement across all recording sessions from a participant. Additionally, we used differential bipolar electrodes for online monitoring of the superficial agonist (Brachioradialis) and antagonist (Triceps Brachii) muscle activities. For all surface sEMG recordings, large reference electrodes (Bagnoli Reference Electrode, Model-SC-R02, Delsys. Inc, electrode diameter = 2 in) were placed over the acromion on the recording side.

### Experimental Protocols

Subjects were positioned in an upright sitting posture on a Biodex chair. Their upper arm joint angles were fixed at elbow flexion 120°, shoulder flexion 10°, shoulder abduction 35°, and the forearm pronation to 45° for maximum activations of biceps muscle during the elbow flexion. To further ensure isolation of the biceps, and thereby reducing contamination of force production from other muscles, the forearm was secured in a custom-made fiberglass orthopedic cast used from above the elbow up to their fingertip. A rigid 6 d-o-f force-torque sensing load-cell (Delta SI-660-60 ATI, NC USA) was mounted on the wrist *via* a metal ring to record generated force. The other end of the load cell was firmly attached to a stable iron platform through fixture arrangements. A rubber pad was placed under the elbow to keep the forearm parallel to the ground. To restrict trunk and shoulder movement a pair of Velcro straps was attached around the subject, to the Biodex chair.

**Figure 1:**
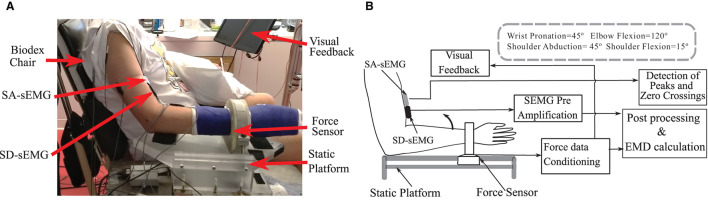
**(A)** The experimental setup and the position details of the force sensor, Single differential (SD-sEMG) sensor and Sensor Array (SA-sEMG) sensors. **(B)** Diagram of the data processing framework.

Voluntary contraction force and sEMG data were recorded during a sustained isometric non-fatiguing elbow flexion task; first, in a maximum voluntary contraction (MVC) trial, then in subsequent trials of submaximal levels at 30% MVC and 50% MVC. Three good trials were performed (with an average error within 10% of the instructed trajectory) at each level with different levels performed in a randomized sequence. During each trial, all forces were simultaneously collected with an sEMG. The resultant force (F_r_) was calculated as the vector summation of the F_x_ and F_z_ recorded from the sensor. Real-time feedback of the force response was provided on a computer monitor. Two-dimensional visual feedback of the force trajectory in the desired quadrant plane assisted the participants to track and to maintain their force profiles as required. Details of the experimental setup and the processing are provided in Figures 1A,B.

Subjects were instructed to follow a two-dimensional trapezoidal force trajectory during the submaximal isometric contraction. The trajectory started with a 5-s rest period, followed by a fixed rate of contraction increase (10% MVC/s), then by the submaximal steady-state contraction level for a period of 10 s, and a decreasing force level at the rate identical to the rate of increment. The average F_r_ during a steady-state force production over a 5-s period was designated as the mean force value. In addition to real-time visual feedback of the instructed trajectory, additional auditory feedback to identify the beginning and the end of the contraction was provided. Subjects were routinely queried regarding fatigue and pain before each trial, and each trial was performed based on the assurance of the subject regarding the same.

### Measurements—EMD: Computer-Based Onset Determination

**Figure 2:**
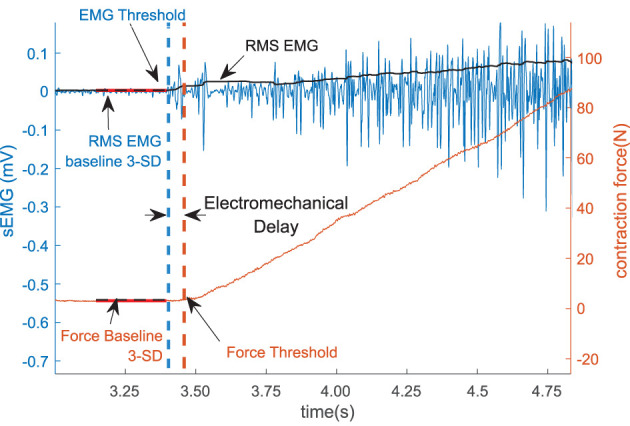
The sEMG and the force threshold. The electromechanical delay (EMD) is denoted as the difference within the sEMG and the force threshold. The sEMG is showed in the left vertical axis and the force is in the right axis.

We define the electromechanical delay as the time delay between the onset of the sEMG and the onset of force production during the increasing phase of the force trapezoid. After the initial qualitative inspection, the method was programmed to automatically detect the onset of force production. The location, where the magnitude of the sEMG trace exceeded a statistical threshold above the background noise level, was determined using a sliding window (50 samples long) as shown in Figure 2.

To reduce the possible effects of noise in the system, 1,000 samples (500 ms) were selected from the sEMG and the force recording channel during the resting period prior to each contraction to determine the respective baseline values. The mean and the standard deviation of the selected resting period activity were calculated, the values for the affected side are summarized in Table 2. The onset of the activity was defined as amplitude greater than 3 standard deviations above the mean resting signal for 25 ms (36).

**Table 2:** The MVC force in the affected (Aff.) and the contralateral (Cnt.) side, the correlation coefficient (CRC) of the force and the EMD values across the sessions in the affected side, and the mean and standard deviation of the RMS sEMG baseline values (mean ± standard deviation) in the affected side.

**Sub**.	**MVC force (Newton)**								**CRC values (affected side)**				**Baseline RMS sEMG (μV) (affected side)**			
	**Pre**		**Post W2**		**Post W6**		**Post W12**		**Pre**	**Post W2**	**Post W6**	**Post W12**	**Pre**	**Post W2**	**Post W6**	**Post W12**
	**Aff**.	**Cnt**.	**Aff**.	**Cnt**.	**Aff**.	**Cnt**.	**Aff**.	**Cnt**.								
B1	30	97	22	102	11	101	27	99	−0.66^*^	−0.72 ^*^	−0.77^*^	−0.81^*^	2.5 ± 0.2	2.9 ± 0.2	2.6 ± 0.5	2.6 ± 0.3
B2	44	116	36	109	37	112	20	115	−0.72	−0.75^*^	−0.63^**^	−0.73^*^	2.3 ± 0.2	2.3 ± 0.2	2.2 ± 0.2	2.2 ± 0.3
B3	36	124	26	118	29	121	26	116	−0.77^*^	−0.54^*^	−0.67	−0.86^*^	2.9 ± 0.4	2.8 ± 0.3	2.9 ± 0.5	2.9 ± 0.5
B4	91	119	26	112	61	121	68	117	−0.84^*^	−0.73^*^	−0.82^*^	−0.81^*^	2.3 ± 0.4	2.3 ± 0.5	2.3 ± 0.5	2.2 ± 0.5
B5	59	177	46	181	57	186	65	182	−0.81^**^	−0.77^**^	−0.83^**^	−0.92^**^	6.5 ± 0.2	5.7 ± 0.2	6.1 ± 0.2	5.9 ± 0.2
B6	65	97	56	99	44	104	–	–	−0.68	−0.42	−0.77^**^	–	2.7 ± 0.3	2.8 ± 0.3	2.7 ± 0.3	–
B7	91	148	68	151	–	–	–	–	−0.77^**^	−0.95^**^	–	–	2.3 ± 0.1	2.7 ± 0.2	–	–
B8	31	55	25	55	–	–	–	–	−0.92^**^	−0.54^**^	–	–	2.8 ± 0.3	2.5 ± 0.2	–	–

* *Indicate p < 0.05,*

** *indicates p < 0.001 and, – indicates the unavailable data*.

The time of the initial sample of the window will be recorded, if the mean of the samples in the window exceeded the threshold. If the mean of the samples in the window did not exceed the criteria, the window was advanced one sample at a time until an onset was found. The time difference between the onset of sEMG and force was determined for both trials at each contraction level.

### Measurements—of ZCs of the sEMG

The ZCs of the sEMG were measured during the steady-state of the force trajectory. The trials for which the EMD were detected were further analyzed for the calculation of ZC. We have only considered one incident of ZC between successive positive and negative peaks detected in sEMG. All the sEMG peaks were detected in a 5-s window (with a minimum variation of the force trace) in the sensor array sEMG signal during the steady-state period for the force trajectory. The region was selected with a minimum force variation of the resulted force trace. This sensor array recording channel, with the highest peak to baseline value, was selected for the peak analysis. The small electrode area and the high recording sampling rates of the sensor array further supported the precise detection of representing the MU firing instances. More detailed descriptions of the sensor can be found in (37). The signal-to-noise ratio (SNR) was calculated for each trial, and recording trials were selected for further analysis when SNR was found to be above 15 dB. In order to characterize the peak amplitude values, a statistically defined EMG threshold was established, peaks detected above this threshold were considered for the distribution analysis. The threshold *E*_*TH*_ was defined as:


$$\begin{array}{l} E_{TH}\;=\;\mu \;\pm \;3\delta \end{array}$$

**Figure 3:**
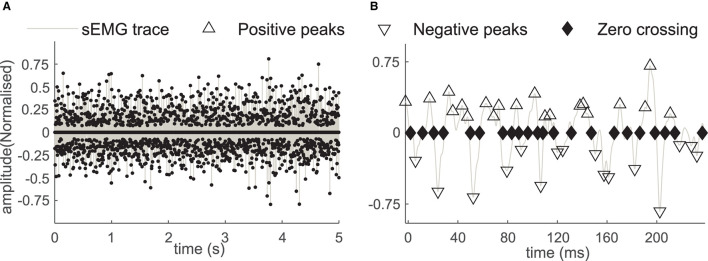
**(A)** The positive and the negative sEMG peaks and the ZC instances in the sEMG during the 5-second window. **(B)** The sEMG and the peaks with a magnified time scale, the positive peaks (marked with Δ), the negative peaks (marked with ∇), and the zero crossings (marked with the ♦) in affected side recording of the subject B2.

Where μ and δ are the mean and the standard deviation of the baseline sEMG signals before the start of the voluntary activation (37). The average number of occurrences of the zero crossings among the trials at the same force levels was calculated as shown in Figure 3.

### Statistical Analysis

The normality of the data was analyzed by the Kolmogorov Smirnov test and the non-normality was confirmed (38). Thereafter, we have used the Kruskal-Wallis test to confirm the statistically significant difference. The null hypothesis was that the samples have been drawn from the same population with an identical median. The null hypothesis was rejected for *p* < 0.05.

## Experimental Results

### EMD Value Differential Between Affected and Contralateral Side During Pre- and Post-W2 Sessions

**Figure 4:**
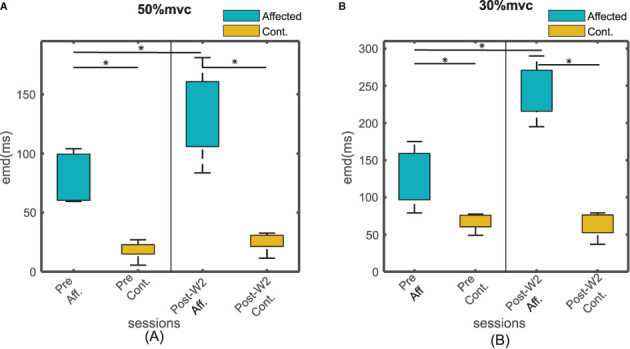
The EMD values for all the subjects for affected and contralateral side, before and after the injection during Post-W2. The sides are plotted with different color during **(A)** 50% and **(B)** 30 % contraction. The box height represents the interquartile range of the EMD values for all the subjects. Statistical significance (*p* < 0.05) is indicated by *.

The EMD values were calculated in both the stroke-affected side and the contralateral side. The EMD values were systematically higher on the affected side (79.7 ± 9 ms at 50% MVC and 127 ± 18 ms at 30% MVC contraction levels) compared to the contralateral side (25 ± 12 ms at 50%MVC and 45 ± 9 ms at 30% MVC contraction levels), as shown in Figures 4A,B. Unlike the affected side, the EMD values in the contralateral side remained relatively unchanged after the BT injection. The maximum variation of the mean EMD values in the contralateral side was found to be within 18% across all the recording session at both contraction levels.

### EMD Values in the Injected Side Across Sessions

**Figure 5:**
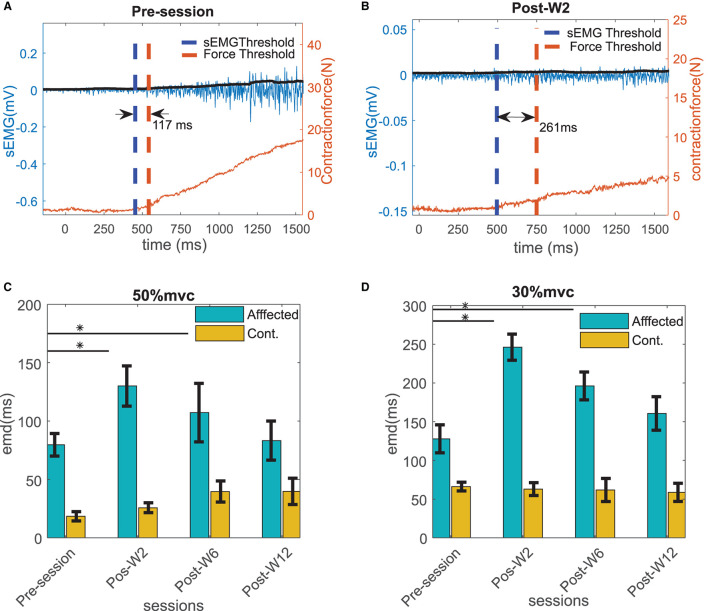
Example of the EMD in the affected side calculated for the subject B2 **(A)** before and **(B)** at Post-W2 after the BT injection shown with the raw sEMG (blue trace with left y scale), rms sEMG (black trace with left y scale), and the force (red trace with right y scale) at 30% contraction level. The thresholds of sEMG and force are marked with blue and red vertical dotted line. **(C)** The electromechanical delays calculated for all the subjects across different sessions at 50 % MVC and **(D)** 30 % MVC contraction trials. The column height represents the mean, while the error bar represents the standard deviation of the ZC values at the session for the subjects. Statistically significant difference of the EMD values in the affected side across sessions (*p* < 0.05) is indicated by *.

Experiments were conducted longitudinally as discussed in the methods section. The EMD values were calculated across the sessions before and after the BT injection. Figures 5A,B show an example of the estimated EMD and the signal traces before and 2 weeks after the injection during the 30% MVC contraction. The EMD values in the affected side showed a statistically significant (*p* < 0.01) increase after the injection (at Post-W2) at both contraction levels compared to their respective pre-injection baseline values. In the affected side, a maximum increase of 64 ± 10 and 93 ± 18% of the EMD values were observed respectively at 50 and 30% MVC levels during Post-W2 compared to the pre-injection values as shown in Figures 5C,D. A significant difference of the EMD values during the post-injection sessions was found compared to the pre session (H_0_ < 0.05, χ^2^ = 7.93). A decaying trend of the EMD values was observed in the following experimental sessions after Post-W2. Finally, at the end of 12 weeks (Post-W12), the EMD values decreased to 7 and 26% for the respective contraction levels as summarized in Figures 5C,D. A strong positive correlation value (*r* = 0.86 ± 0.04 with *p* < < 0.05) was found among the time course of the EMD values at two contraction levels.

### Variations in the Contraction Forces Across Sessions

The maximum voluntary contraction (MVC) force values, and, thus, the submaximal contraction values are varied across the post-BT recording sessions. Table 2 summarizes the MVC levels for all the subjects across the session. Part of the force values have been restated from our previous work for clarity purposes (13). In general, the contraction force values across the recording sessions decreased in the sessions just after the BT injection, then gradually increased to sub-baseline levels. An average reduction of 30% in the contraction force was observed compared to their pre-injection baseline values. However, the force values recorded at Post-W3 and W4 were often larger than those recorded at Post-W2, which could be characterized as the minimum in the recorded force time series. The pattern of the recovery from this minimum value varied across the subjects as noted in Table 2. The EMD values were lower at higher contraction levels for all the subjects. This phenomenon was observed for both the BT injected affected side, as well as the uninjected contralateral side. The EMD values in the affected side at 30% MVC is 45 ± 21% higher than the value at 50% MVC, while on the contralateral side the value was 35 ± 18 % (as shown in Figures 5C,D). The Pearson correlation coefficient between the EMD values and the force values were separately calculated at different contraction level, while negative correlation coefficients were observed for all the subjects. The correlation coefficients among the contraction force and the EMD values for the pre- and post-W2 sessions in the affected side are summarized subject wise in Table 2.

### The Force-EMD Slopes Across Sessions

**Figure 6:**
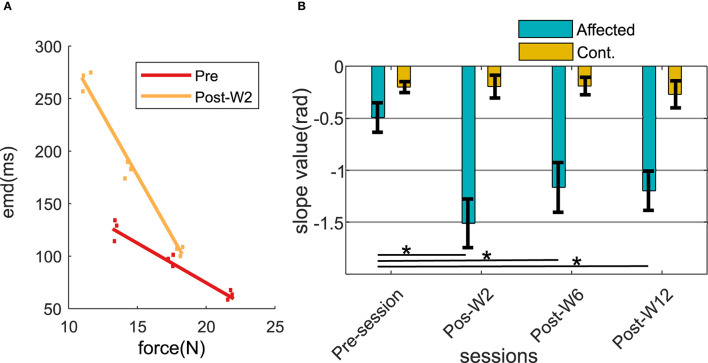
**(A)** Force-EMD regression slope of subject B2 during Pre- and Post-W2 session in the affected side. **(B)** Slope value across different experimental sessions for all the subjects represented in radians. The column height represents the mean, while the error bar represents the standard deviation of the ZC values at the session for the subjects. Statistically significant difference of the EMD values in the affected side across sessions (*p* < 0.05) is indicated by *.

The force and the EMD values were measured respectively during 30, 40, and 50% of the MVC level across the experimental sessions. After the injection, a linear regression line was fitted (min *r* > 0.68) within the force and the EMD values. In order to assess the changes in the relation between the force and the EMD, the slope of the linear fit was analyzed across the experimental sessions. The slope values of the linear fit were found to systematically vary in the injected side for all the subjects as shown in Figures 6A,B.

During the pre-injection recording session, the slope values in the injected side (−0.49 ± 0.16) were found to be lower than the contralateral side (−0.22 ± 0.07). At Post-W2, the slope value in the affected side showed a statistically significant (*p* = 0.0113) reduction of 3.5 times compared to the pre-injection values. The reduced slope value persisted until 12 weeks for the subjects (*p* = 0.006) compared to its pre-injection slope value. Despite the increase of the slope values compared to the post W2 were observed from Post-W6 onward, even at the end of 12 weeks (Post-W12), the values were found to be significantly lower than the pre-injection value recorded at −1.19. During this session, the slope values in the contralateral side remained high in all the experimental recording session across the time course, and the variation recorded were within 20% of all the contralateral values recorded among the subjects.

### Characterization of the sEMG Zero-Crossings Across Sessions

**Figure 7:**
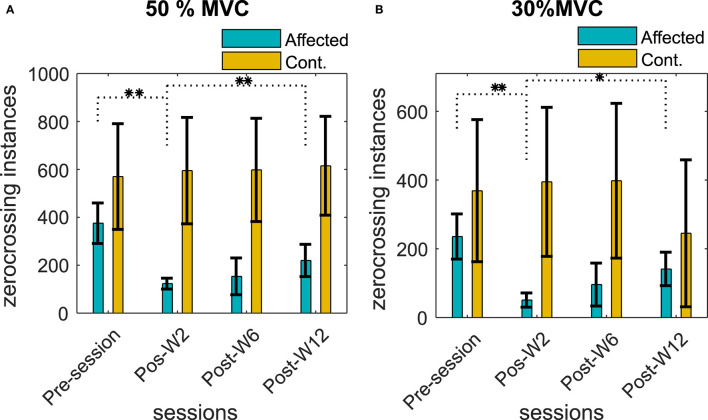
Number of ZCs in the sEMG signal during **(A)** 50% MVC and **(B)** 30% MVC contraction level across the recording sessions. The column height represents the mean, while the error bar represents the standard deviation of the ZC values at the session for the subjects. Statistically significant difference of the EMD values (*p* < 0.05/*p* < 0.01) in the affected side across sessions is indicated by */**.

The number of ZCs was found to be directly proportional to the contraction level, the 50% contraction level had the highest number of ZC, and the number of ZC during the 30% contraction was the lowest. The number of ZCs on the affected side before the injection was found to be lesser (<30%) than the contralateral side for all the subjects when compared to the respective contraction level. Figures 7A,B summarize the number of zero crossings of all the subjects across different recording sessions before and after the BT injection at 50 and 30% MVC contraction levels.

The number of ZCs in the affected side was reduced after the BT injection, and the average ZCs among the subjects were found to be lower than the pre-injection baseline recording. The reduction of the number of ZCs was statistically significant (*p* < < 0.05) for the test cohort.

The average reduction of ZC was 50 ± 16, 74 ± 24, and 18 ± 11% at Post-W2, Post-W6, and Post-W12, respectively, compared to the pre-injection value of the respective subjects. The number of ZCs did not return to the baseline pre-injection values when compared individually for the subjects who have studied for full 12 weeks apart from B5. Unlike the injected side, the number of ZCs across the sessions in the uninjected contralateral side remained relatively unchanged with a statistical significance (*p* > 0.08) for all the subjects at all contraction levels.

Along with the ZC values, the linear regression slopes between the contraction force and the ZC relations were also analyzed. We found a reduction in the force-ZC slope values after the injection in all our subjects (except B5). The average reduction in the slope values for the subjects at Post-W2 was 38 ± 31% compared to the Pre-session. The force-ZC slope values did not recover to the pre-injection baseline level by Post-W12. While the systematic reduction of the force-ZC was observed in the affected side, the contralateral side values did not change significantly, and no systematic difference was found.

## Discussion

The goal of this study was to investigate the time-course of the EMD during isometric voluntary contraction in spastic biceps muscle followed by a clinically prescribed injection in the muscle of our tested cohort. Our results show that estimating EMD might provide an insight into the changes in neural control—i.e., changes in recruitment of motor units due to a BT-induced chemodenervation in spastic biceps muscle. We have measured the EMD between the sEMG activation and the resulting detectable elbow contraction force at various levels of contraction. Simultaneously, we have studied the number of ZCs in the sEMG signal based on a novel sensor array recording. Recordings from the contralateral side were used as a control reference (i.e., uninjected muscle) for the experimental analyses.

### Systematic Changes in Force and EMD

The primary finding of this study was that the EMD values increase by 64 ± 10% (at 50% MVC) and 93 ± 18% (at 30% MVC), respectively, during the first recording session after the BT (Post-W2) for our test cohort, while the mean force value was also reduced by 8%. We have found maximum EMD values that occurred during the first post-injection session (Post-W2). Thereafter, the values declined gradually during the following sessions in the later stage (at Post-W6 and W12). Concurrently, the force also partially recovers back from its minimum value among four of the six subjects (except B3 and 4) who were studied for additional sessions after the initial session (Post-W2).

Despite the partial recovery of the following sessions, there exists a 7 and 15% prolongation in the EMD values during session Post-W12 compared to the Pre-session baseline as shown in Figures 5C,D. We have also found that the EMD values were inversely proportional to the contraction level for all the subjects in our test cohort; the measured value at 30% MVC is 45 ± 21% higher than the value at 50% MVC. The driving hypothesis behind this study was that due to the BT-induced chemodenervation of the fibers, the limited availability of the active fibers in an MU results in a reduced twitch strength (39). Additionally, a complete denervation of all the fibers from the associated motor neuron (MN) results in an absence of twitch. These two phenomena collectively facilitate suboptimal twitch superposition (40), resulting in an inefficient force production during the voluntary contraction. As a result, delays are increased during the overall force production process. This effect of BT in muscles has been earlier speculated by (41), in addition to the functional deficits attributable to changes in muscle architecture and in mechanical properties (42).

The highest antispastic effect of the BT induces the denervation found within 1 month of the injection affecting the contraction force and the sEMG. This has been reported earlier by several groups including us (13, 43).

We have observed higher standard error in the EMD values at lower force levels. This is potentially due to the variation in the effect of BT in the muscle as suggested by earlier groups (44). A major contributing factor in this scenario is the length of the muscle-tendon unit (5), which is often affected by prolonged dosages of BT (15). The trend of the EMD values was well correlated with the MVC force for our subjects across the different sessions. We have mentioned the CRC for the pre- and the post-sessions as listed in Table 2. We have found that the CRC for all sessions were having a negative value. The contraction force-dependent nature of EMD values, as we have observed during the experiment, matches with the conclusions of (45). During this session, the EMD values in the contralateral side were significantly less (<65 ms) compared to the respective affected side. This also indicates a higher number of available fibers in the contralateral muscle.

### Zero Crossings as an Indication of Reduced MU Activity

Since a functional alteration of the MU pool will be reflected in the sEMG, we have used the zero-crossings value of the sEMG signal as a surrogate of the recruited MU (28, 30). The increased EMD has been earlier identified as a function of the recruited active MU (4). The influence of neural control on EMD has also been recently highlighted by Schmid et al., in which the authors concluded that the larger EMD during voluntary contraction is the result of an underlying MU recruitment and firing strategies (2) which are in agreement with our result.

Our results show a dramatic reduction in the number of ZCs among our subjects that may signify a reduced number of active fibers. A reduction of active fibers after BT injection was reported earlier (15). We have observed a higher number of ZC (1.7 ± 0.8 times) during the 50% MVC compared to the 30% MVC, suggesting an increased number of MU activity at higher force levels. Our result suggests that during Post-W2, when the EMD reached its maximum value, the ZC reaches the minimum. During the Post-W6 and Post-W12, the ZC value increases while the EMD values were found to be decreasing. This corroboration indicates the potential influence of the altered MU function on EMD.

Reduction in the force-ZC slopes provides a means of comparing the ZC despite the reduction of the contraction force during the post BT session to the respective pre-injection baseline values. Reduction of the force-ZC slope at Post-W2 indicates a disproportionate reduction of the motor outflow compared to the reduction in force in the BT-injected muscle.

Disproportionately larger reductions of contraction force may have two reasons: (1) A change in series elasticity of the fascicle due to partial denervation of muscle fibers within the fascicle after BT injection (46), and (2) a disorganized recruitment and firing of the MU resulting in an inefficient twitch summation (47). Moreover, these factors are related to each other, since earlier studies have reported a drastic impact of series elasticity on the rate of force rise and doublet force summation (48).

In order to understand the relation between EMD and the neural drive to the active MN pool, we have studied the force EMD slopes. The negative slope values for all the subjects across all the recording sessions indicate a negative correlation between the MVC levels and the EMD values (as shown in Table 2). We have found a reduction in the slope inclination value (>200%) after the injection (Figures 6A,B). Higher slope values were contributed by the larger differential of the EMD values compared to the respective differential in force. Potentially, this may be related to the reduced number of the available fibers after the chemodenervation that induces deviation from the original recruitment rank order of the MN pool. An altered MN pool, as earlier suggested by Vint et al. (4) Hopkins et al. (49), gives rise to the earlier recruitment of the available MU during the higher (50% MVC) and the lower (30% MVC) contraction level. While the slope value in the injected side was reduced 3.5 ± 0.4 times in the affected side, no statistically significant difference has been found in the slope values in the uninjected side across the recording session.

It is important to mention that at the end of 3 months, the Force-EMD slope value did not come back to the pre-injection level, while no significant differences were evident in the EMD values (for B1–B5) compared to their pre-injection level. This may be attributable to the architectural limitations of the shortcoming in a spastic muscle during the contraction, which also played an important role in the force generation (47). Moreover, the stiffness of the spastic muscle has also been reported to be influenced by a BT injection, and this could also influence the force generation (5).

### Limitations

Computation of the EMD critically depends upon the calculation method and the nature of the muscle activation task. We have defined EMD as a time delay between the detected onset of the sEMG and the respective voluntary contraction force (36). Existing variation of an earlier reported EMD is evident, and it depends upon the process of the threshold calculation. Moreover, there are other factors that are reported to be an imposed variability on the EMD data. However, in our study, the contralateral EMD values closely match with the earlier reported values < 50 ± 45 ms (8, 50), while the affected side shows a higher value, perhaps, due to the reduced number of available fibers and architectural limitation of the muscle. Potentially, the EMD values can be influenced by the characteristics of the baseline signal standard deviation, which are calculated when the muscle was at rest. However, we did not find any significant or systematic difference in the standard deviation values of the baseline sEMG signal as noted in Table 2. We have calculated the number of zero-crossings based on the detected peak pairs (positive-negative) in the channel where the largest number of peaks were observed. This process is dependent on the detected peaks. Detection of sEMG peaks during the steady-state contraction depends on the baseline noise of the sEMG. We have deducted the noise value recorded at the beginning of each trial from the signal during the steady-state. The subject cohort reported in this study are heterogeneous in terms of sex, post-stroke time, BT naivety, and dosage that may influence the result. With a smaller cohort size, we have attained limited power of our findings. However, the drastic changes of the EMD were evident despite these factors. A limitation of our study was the potential activation of the flexion synergist during the isometric flexion task, thereby potentially reducing the EMG recorded from the biceps. In order to minimize these effects, upper arm joint angles were carefully selected for maximum activation of biceps, which were maintained at each session for each subject to reduce the activation. Simultaneous recording from the synergist was used during the experiment to ensure minimum activation of the other muscle before the trial.

## Conclusion

EMD is, in part, a measure of functional architecture and internal disruption of the muscle. This study provides a quantitative characterization of changes in the EMD and the number of zero-crossing in the sEMG signal after the intramuscular BT injections in stroke survivors, which allowed us to understand the effects of BT more clearly on voluntary contraction. The results suggest that the BT-induced reduction of spasticity results in a reduction in voluntary contraction capacity in association with the increased EMD value. The increase in EMD indicates that the BT disrupted both the fascicle architecture and the normal mechanics of force production that contributed to the clinical impairment. We have further shown the increase in EMD is at least, in part, contributed by the reduced number of firings, which was measured in terms of the zero-crossing in the sEMG signals, and does not come back to its pre-injection baseline after 12 weeks. The result of this study is the first evidence of a change in EMD affecting the force production capacity after the BT injection.

## Data Availability Statement

The datasets used and/or analyzed during the current study are available from the corresponding author on reasonable request.

## Ethics Statement

The studies involving human participants were reviewed and approved by the Institutional Review Board at Northwestern University (IRB No. STU00200242). The patients/participants provided their written informed consent to participate in this study. Written informed consent was also obtained from the individual(s) for the publication of any potentially identifiable images or data included in this article.

## Author Contributions

SC, BA, WR, and NS participated in the study conception, design, data analysis, and interpretation. SC, BA, and NS participated in data acquisition. All authors read and approved the final manuscript.

## Funding

This study was funded by the National Institute on Disability, Independent Living, and Rehabilitation Research (NIDILRR-90RE5013) through Advanced Rehabilitation Research and Training (ARRT) and Rehabilitation Engineering Research Center (RERC) program.

## Conflict of Interest

The authors declare that the research was conducted in the absence of any commercial or financial relationships that could be construed as a potential conflict of interest.

## Publisher's Note

All claims expressed in this article are solely those of the authors and do not necessarily represent those of their affiliated organizations, or those of the publisher, the editors and the reviewers. Any product that may be evaluated in this article, or claim that may be made by its manufacturer, is not guaranteed or endorsed by the publisher.
